# Composition-Driven Surface Reorganization and Fractal Scaling in PCL/BaMTi^4+^ Polymer–Ferrite Composite Films

**DOI:** 10.3390/polym18172059

**Published:** 2026-08-25

**Authors:** José Victor Bezerra Teixeira, Wisley Prata Lima, Célio dos Santos Almeida, Fidel Guerrero Zayas, Ştefan Ţălu, Robert Saraiva Matos, Carlos Alberto Rodrigues Costa, Marcos Marques da Silva Paula, Henrique Duarte da Fonseca Filho

**Affiliations:** 1 Laboratório de Materiais Nanoparticulados (LAMAN), Departamento de Física de Materiais, Universidade Federal do Amazonas, Manaus 69067-005, AM, Brazil; jose.teixeira@ufam.edu.br (J.V.B.T.); wisley.prata@ufam.edu.br (W.P.L.); celioalmeida08@gmail.com (C.d.S.A.); fgzayas@gmail.com (F.G.Z.); marcosmarques@ufam.edu.br (M.M.d.S.P.); 2 The Directorate of Research, Development and Innovation Management (DMCDI), Technical University of Cluj-Napoca, Constantin Daicoviciu St., no. 15, 400020 Cluj-Napoca, Romania; stefan.talu@auto.utcluj.ro; 3 Grupo de Materiais Amazônicos, Departamento de Física, Universidade Federal do Amapá, Macapá 68903-419, AP, Brazil; robert_fisic@unifap.br; 4 Laboratório Nacional de Nanotecnologia (LNNano), Centro Brasileiro de Pesquisa em Energia e Materiais (CNPEM), Campinas 13083-100, SP, Brazil; carlos.costa@lnnano.cnpem.br; 5 Laboratório de Desenvolvimento e Aplicações de Nanomateriais da Amazônia (LADENA), Departamento de Física de Materiais, Universidade Federal do Amazonas, Manaus 69067-005, AM, Brazil

**Keywords:** poly(ε-caprolactone), Ti^4+^-doped barium hexaferrite, polymer–ferrite composites, AFM topography, fractal scaling

## Abstract

Flexible polymer–ferrite composites provide a route for lightweight functional materials with tunable surface and structural properties. Here, poly(ε-caprolactone) (PCL)/Ti^4+^-doped barium hexaferrite (BaMTi^4+^) films containing 10–50 wt.% ferrite were prepared by solvent casting and characterized by XRD, FTIR, SEM, AFM, and fractal analysis. XRD confirmed the coexistence of semicrystalline PCL and magnetoplumbite-type BaMTi^4+^, whereas FTIR indicated preservation of the polymer backbone and non-covalent interfacial interactions. Morphological and topographical analyses revealed a composition-dependent transition from compact polymer-rich surfaces to rougher ferrite-rich architectures. Surface roughness decreased at 10 wt.% BaMTi^4+^ and increased at higher loadings, reaching Sa = 33.10 nm and Sq = 42.54 nm at 50 wt.%. Power spectral density and fractal analyses showed enhanced spatial correlation, with the Hurst exponent increasing from 0.51 to 0.81 and the fractal dimension decreasing from 2.49 to 2.19. These results demonstrate that ferrite loading effectively controls the multiscale surface organization of magnetically active PCL films.

## 1. Introduction

**Figure 1:**
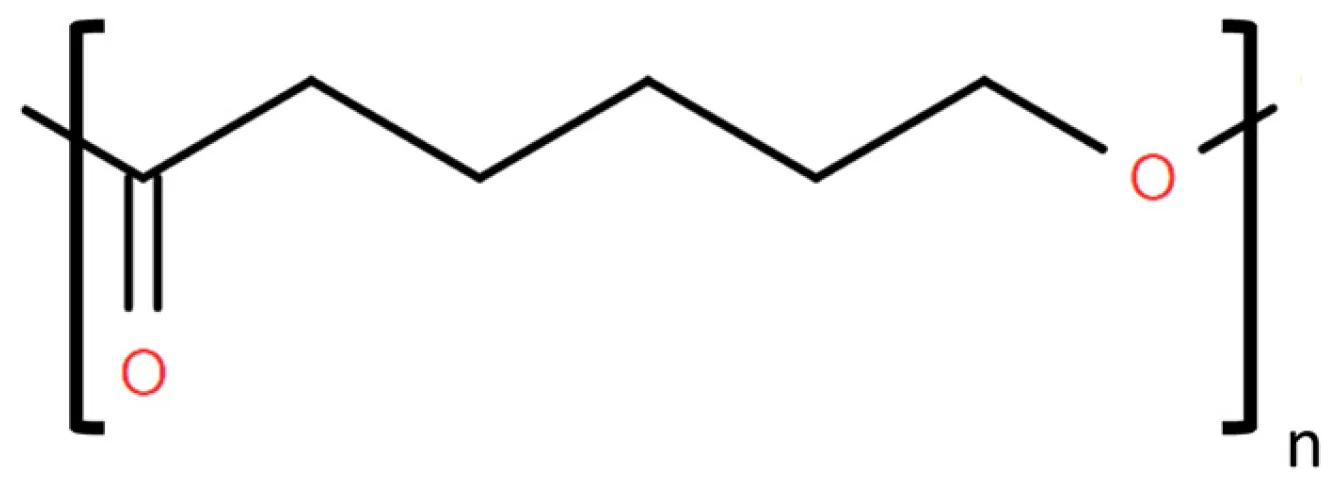
Chemical structure of the repeating unit of poly(ε-caprolactone) (PCL).

The development of flexible polymer–ceramic composites has become a central strategy in materials physics for engineering lightweight, processable, and multifunctional films with tunable structural, interfacial, magnetic, dielectric, and electromagnetic responses. Unlike conventional monolithic ceramics, polymer–ceramic hybrids offer mechanical compliance, low density, shape adaptability, and scalable fabrication while retaining functional contributions from the inorganic phase [1,2,3,4]. This design concept is particularly attractive for next-generation electromagnetic, biomedical, sensing, energy-storage, and shielding platforms, where performance is increasingly governed not only by phase composition but also by filler dispersion, interfacial coupling, surface morphology, and multiscale topographical organization [5,6,7,8]. Recent advances in polymer-based electromagnetic interference shielding and microwave-absorbing composites have emphasized that efficient attenuation requires a delicate balance among dielectric loss, magnetic loss, impedance matching, interfacial polarization, and microstructural continuity rather than a simple increase in filler concentration [9,10,11,12]. Poly(ε-caprolactone) (PCL), with the repeating unit formula (C_6_H_10_O_2_)_n_ and chemical structure shown in Figure 1, is a semicrystalline aliphatic polyester widely explored as a matrix for hybrid materials because of its biodegradability, flexibility, film-forming ability, low processing temperature, and compatibility with inorganic particles [13,14,15].

Its semicrystalline nature is especially relevant from a physical standpoint: the final arrangement of PCL chains depends on crystallization kinetics, solvent evaporation, lamellar growth, and chain mobility during solidification [16,17,18]. In composite systems, these processes are further affected by particle surfaces, which can either promote heterogeneous nucleation or impose local confinement that restricts chain diffusion and lamellar thickening [19,20]. Thus, the addition of inorganic particles to PCL does not merely introduce a second phase; it changes the thermodynamic and kinetic conditions under which the polymer matrix organizes. Understanding this competition between nucleation, confinement, and interfacial restriction is essential for designing PCL-based composites with predictable structure–property relationships.

Magnetic ferrites are particularly attractive inorganic fillers because they combine chemical stability, magnetic response, dielectric activity, and electromagnetic attenuation capability [20,21,22]. Among them, M-type barium hexaferrite (BaFe_12_O_19_) crystallizes in the hexagonal magnetoplumbite structure with space group P6_3_/mmc. Its strong magnetocrystalline anisotropy, high coercivity, and robust magnetic behavior make it suitable for permanent magnets, microwave absorption, electromagnetic shielding, and functional polymer composites [21,22,23].

The functional properties of barium hexaferrite can be further tuned through cationic substitution at Fe sites, which modifies local charge distribution, defect chemistry, anisotropy, and exchange interactions [24,25].

In this regard, Ti^4+^-doped barium hexaferrite (BaFe_12−x_Ti_x_O_19_, hereafter referred to as BaMTi^4+^) is an attractive magnetic ceramic phase for polymer composites because Ti^4+^ substitution provides a route for tailoring the structural, magnetic, and dielectric properties of BaM. As Ti^4+^ is a non-magnetic ion, its incorporation into Fe sites modifies the Fe^3+^–O^2−^–Fe^3+^ superexchange interactions and can reduce the magnetocrystalline anisotropy, coercivity, and saturation magnetization. Because substitution of Fe^3+^ by Ti^4+^ requires charge compensation, changes in the Fe^3+^/Fe^2+^ balance and associated electron-hopping processes may also affect the electrical conductivity and dielectric response. In addition, Ti^4+^ substitution has been reported to modify the natural ferromagnetic resonance and impedance-matching conditions of barium hexaferrite, which are relevant to microwave absorption and electromagnetic attenuation. Therefore, Ti^4+^ doping provides a means of tuning the coupled magnetic and dielectric response of BaM for its incorporation into functional polymer–ceramic composites [26,27,28,29].

Despite these advantages, incorporating ferrite particles into semicrystalline polymer matrices remains scientifically challenging. At low filler contents, ferrite particles may serve as discrete nucleation centers, redistributing polymer-chain organization and stabilizing local crystalline domains. At high filler contents, however, particle–particle proximity may induce aggregation, increase interfacial confinement, reduce polymer-chain mobility, and generate heterogeneous stress fields within the film [30,31]. These effects can strongly influence not only crystallinity and morphology but also the surface architecture that controls adhesion, wettability, interfacial polarization, and mechanical integrity. Therefore, the functional behavior of PCL/ferrite composites should not be interpreted solely in terms of filler loading. Instead, it must be understood through the coupling among phase coexistence, polymer crystallization, filler dispersion, and the topological structure of the polymer–ceramic interface [32,33]. However, a key limitation in many polymer–ferrite studies is that morphology is often discussed qualitatively or roughness is reduced to a single average value. However, surfaces are inherently multiscale physical systems. Average roughness parameters such as Sa and Sq quantify the vertical amplitude of height fluctuations, but they do not reveal whether the surface is dominated by isolated asperities, valley-rich domains, short-wavelength disorder, or long-range correlated features [33,34]. Likewise, skewness and kurtosis provide essential information on height asymmetry and the statistical sharpness of the surface distribution, which are relevant for contact mechanics and bearing behavior [35]. Power spectral density analysis and fractal descriptors go further by resolving how roughness is distributed across spatial frequencies, allowing the surface to be interpreted in terms of scale-dependent organization, spatial persistence, and fractal complexity [32,34]. Such an approach is particularly valuable for polymer–ceramic films, where roughness may arise from multiple competing mechanisms, including solvent-casting dynamics, polymer crystallization, particle dispersion, and filler aggregation.

Recent studies on PCL-based magnetic and ceramic composites have demonstrated that the incorporation of inorganic particles can modify mechanical response, degradation behavior, magnetic functionality, and surface morphology [16,36,37]. Magnetic Fe_3_O_4_/PCL scaffolds, for example, have shown that particle size and concentration influence scaffold performance and magnetic response [14,36]. PCL composites containing metallic or ceramic fillers have also shown that filler dispersion and interfacial compatibility are decisive for determining mechanical and topographical behavior [38,39]. In parallel, ferrite–polymer systems have been increasingly explored for energy storage, electromagnetic shielding, and microwave absorption, where functional performance depends strongly on the continuity of magnetic/dielectric pathways and the quality of the polymer–ferrite interface [4,40]. These studies collectively indicate that a physically rigorous description of PCL/ferrite films requires simultaneous consideration of crystalline phase preservation, chemical integrity, mesoscale morphology, and nanoscale surface scaling. However, despite these advances, the literature has not yet provided a systematic surface-physics description of PCL films loaded with Ti^4+^-doped barium hexaferrite in which phase structure, interfacial preservation, mesoscale morphology, areal roughness, height-distribution statistics, bearing area behavior, and PSD/fractal scaling are treated as interconnected descriptors of the same material architecture. Most available studies emphasize filler incorporation, magnetic response, biological performance, or average roughness, but they rarely resolve how increasing ferrite loading reorganizes the polymer surface across multiple spatial scales. This gap is important because the functional behavior of polymer–ferrite composites is expected to emerge not only from the presence of the magnetic ceramic phase but also from the way its interfaces constrain polymer-chain mobility, reshape crystalline domains, and generate correlated surface features.

Harnessing the structural robustness of Ti^4+^-doped barium hexaferrite ($Ba{Fe}_{11.9}O_{19}{Ti}_{0.1}$) and the processability of semicrystalline PCL, we engineer magnetically active free-standing PCL/BaMTi^4+^ polymer–ceramic films through a solvent-casting route that enables controlled ferrite loading and interfacial organization. This approach reveals how BaMTi^4+^ content reshapes the crystalline, chemical, morphological, and topographical hierarchy of the films, driving a transition from polymer-controlled organization to ferrite-rich surface architecture. By connecting phase coexistence, polymer-chain preservation, mesoscale heterogeneity, and fractal surface scaling, this work establishes a physically grounded framework for designing flexible PCL/ferrite composites in which functional performance can be tuned through composition, interfacial coupling, and nanoscale surface organization.

## 2. Materials and Methods

**Figure 2:**
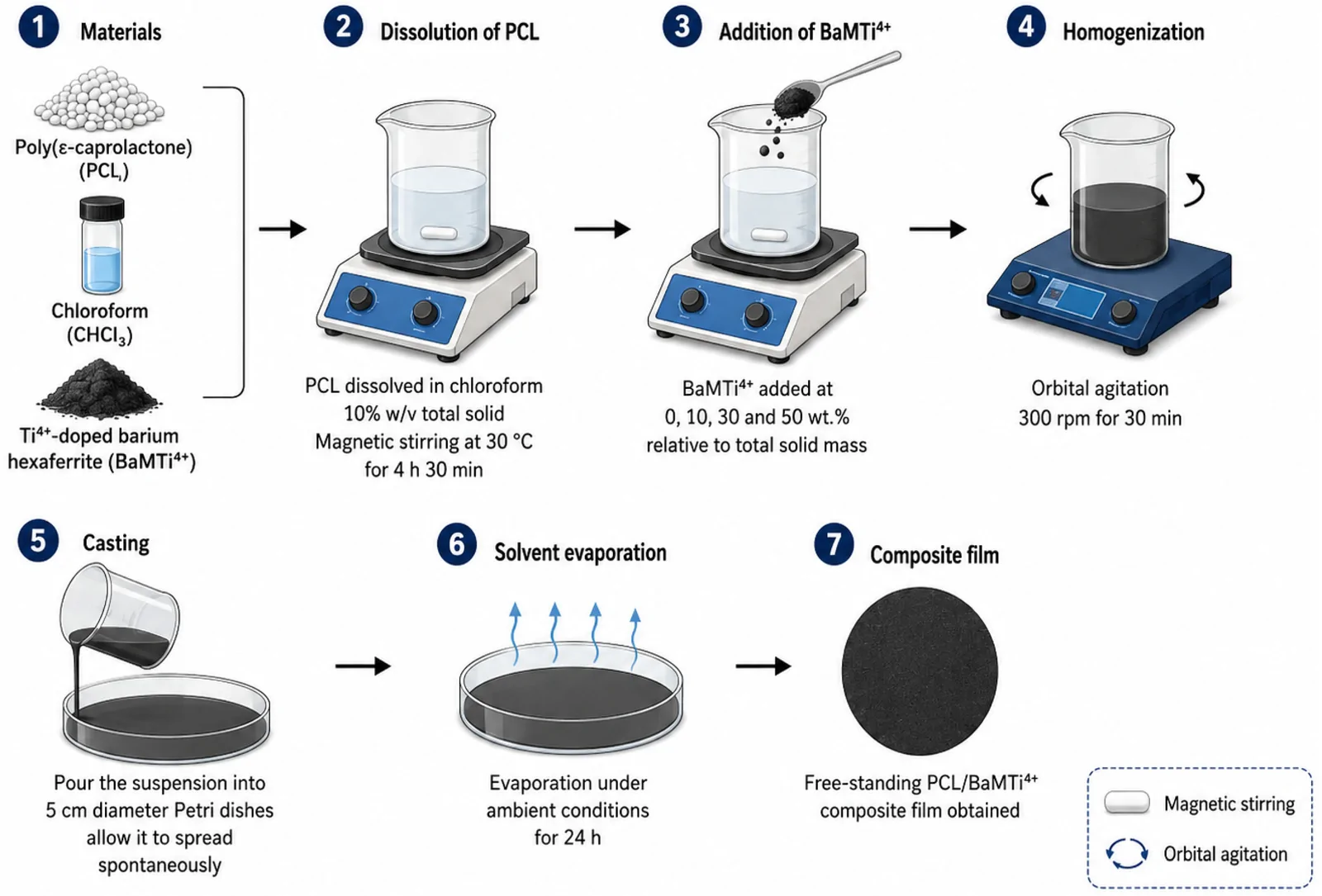
Schematic illustration of the solvent-casting route for the preparation of PCL/BaMTi^4+^ composite films.

PCL/BaMTi^4+^ composite films were prepared by solvent casting [1], as schematically illustrated in Figure 2. PCL was obtained from Sigma-Aldrich (St. Louis, MO, USA; Mn = 80,000). Chloroform was obtained from Nuclear (Angra dos Reis, Brazil; analytical grade) and used as received. In a typical procedure, poly(ε-caprolactone) (PCL) was first dissolved in chloroform to obtain a 10% (*w*/*v*) total solid concentration. The total mass of solid components was fixed at 0.50 g in 5 mL of chloroform. The PCL/chloroform solution was magnetically stirred at 30 °C for 4 h 30 min to ensure complete polymer dissolution and formation of a homogeneous solution. After PCL solubilization, Ti^4+^-doped barium hexaferrite powder (BaMTi^4+^), synthesized previously by a coprecipitation route reported in [29], was incorporated into the polymer solution at nominal contents of 10, 30, and 50 wt.% relative to the total solid mass. The corresponding PCL/BaMTi^4+^ mass ratios were 0.50/0.00, 0.45/0.05, 0.35/0.15, and 0.25/0.25 g, respectively, as summarized in Table 1. The resulting composite films were designated as PCL/BaMTi^4+^-10, PCL/BaMTi^4+^-30, and PCL/BaMTi^4+^-50, where the numerical suffix denotes the BaMTi^4+^ content (wt.%) relative to the total solid mass. The polymer-only sample is hereafter referred to as neat PCL. The resultant PCL/BaMTi^4+^ suspensions were homogenized under magnetic agitation at 300 rpm for 30 min to promote dispersion of the ferrite particles within the polymer solution. The mixtures were then poured into Petri dishes with a diameter of 5 cm and allowed to spread spontaneously over the substrate surface. Solvent evaporation was carried out under ambient conditions for 24 h. After drying, the free-standing films were carefully detached from the Petri dishes and stored in a desiccator before investigation.

### 2.1. Characterization

The crystalline structure of the PCL/BaMTi^4+^ composite films was investigated by X-ray diffraction using a Shimadzu Maxima XRD-7000 diffractometer (Shimadzu Corporation, Kyoto, Japan) equipped with Cu Kα radiation (λ = 1.5406 Å). The measurements were carried out at an operating voltage of 40 kV and a current of 30 mA. Initial scans were collected over the 2θ range of 5–100° to verify the overall diffraction response of the samples. For detailed phase analysis, diffractograms were recorded from 10° to 80°, using a step size of 0.02° and a scanning rate of 2° min^−1^. Chemical structure and possible interfacial interactions between PCL and BaMTi^4+^ were assessed by Fourier-transform infrared spectroscopy in attenuated total reflectance mode (FTIR-ATR). The spectra were acquired in the 4000–500 cm^−1^ range using an Agilent Cary 630 spectrometer (Agilent Technologies, Santa Clara, CA, USA) equipped with a ZnSe ATR crystal. The thickness of the dried films was measured using a digital thickness gauge with a measurement range of 0–25.4 mm and a resolution of 0.01 mm. Ten measurements were taken at different positions across each film, and the results are reported as mean ± standard deviation. Surface morphology was examined by scanning electron microscopy (SEM) using a JSM-6390LV microscope (JEOL Ltd., Akishima, Tokyo, Japan) operating at 15 kV. The samples were metallized with Pt using a sputtering process. To ensure electrical conductivity, the samples received a platinum coating via sputtering (LEICA, EM ACE600). Atomic force microscopy (AFM) measurements were performed in tapping mode at room temperature over scan areas of 10 × 10 µm^2^. To quantify the amplitude, asymmetry, and statistical distribution of the surface heights, areal topographical parameters were extracted from the AFM height maps according to ISO 25178-2:2012, including root-mean-square height (Sq), arithmetic mean height (Sa), skewness (Ssk), and kurtosis (Sku) [41,42,43]. To evaluate the spatial organization and scaling behavior of the film surfaces, power spectral density analysis was performed using the AFM topographic data. The one-dimensional PSD was calculated from the height profile according to Equation (1) [34]:

(1) $${PSD}^{1D}\left(q_{x}\right)=L_{x}^{-1}{\left[\int_{L_{x}}h(x,y)e^{-iq_{x}x}dx\right]}^{2}$$

where L_x_ is the profile length, h(x,y) is the surface height extracted from the AFM topographic map, and q_x_ is the spatial wave vector. The linear region of the log–log PSD curves was used to determine the scaling exponent α, which describes the decay of roughness amplitude as a function of spatial frequency. The Hurst exponent (H) was calculated from the PSD slope using Equation (2) [34]:

$$H=\frac{\alpha -2}{2}$$

The fractal dimension D_f_, which describes the degree of surface complexity, was then obtained as D_f_ = 3 − H, as commonly applied for self-affine three-dimensional surface topographies [32,34]. Higher H values indicate more spatially correlated and persistent surface features, whereas higher D_f_ values indicate greater topographical complexity and more irregular surface fluctuations. This approach allowed the roughness amplitude parameters to be complemented by scale-dependent descriptors of surface organization in our PLC/BaMTi^4+^ system.

### 2.2. Statistical Analysis

Statistical analysis was performed using one-way analysis of variance (ANOVA) to evaluate significant differences among the sample groups. When significant differences were detected, Tukey’s post hoc test was applied for pairwise comparisons. Differences were considered statistically significant at *p* < 0.05. Data are reported as mean ± standard deviation.

## 3. Results

### 3.1. Structural and Chemical Analysis

**Figure 3:**
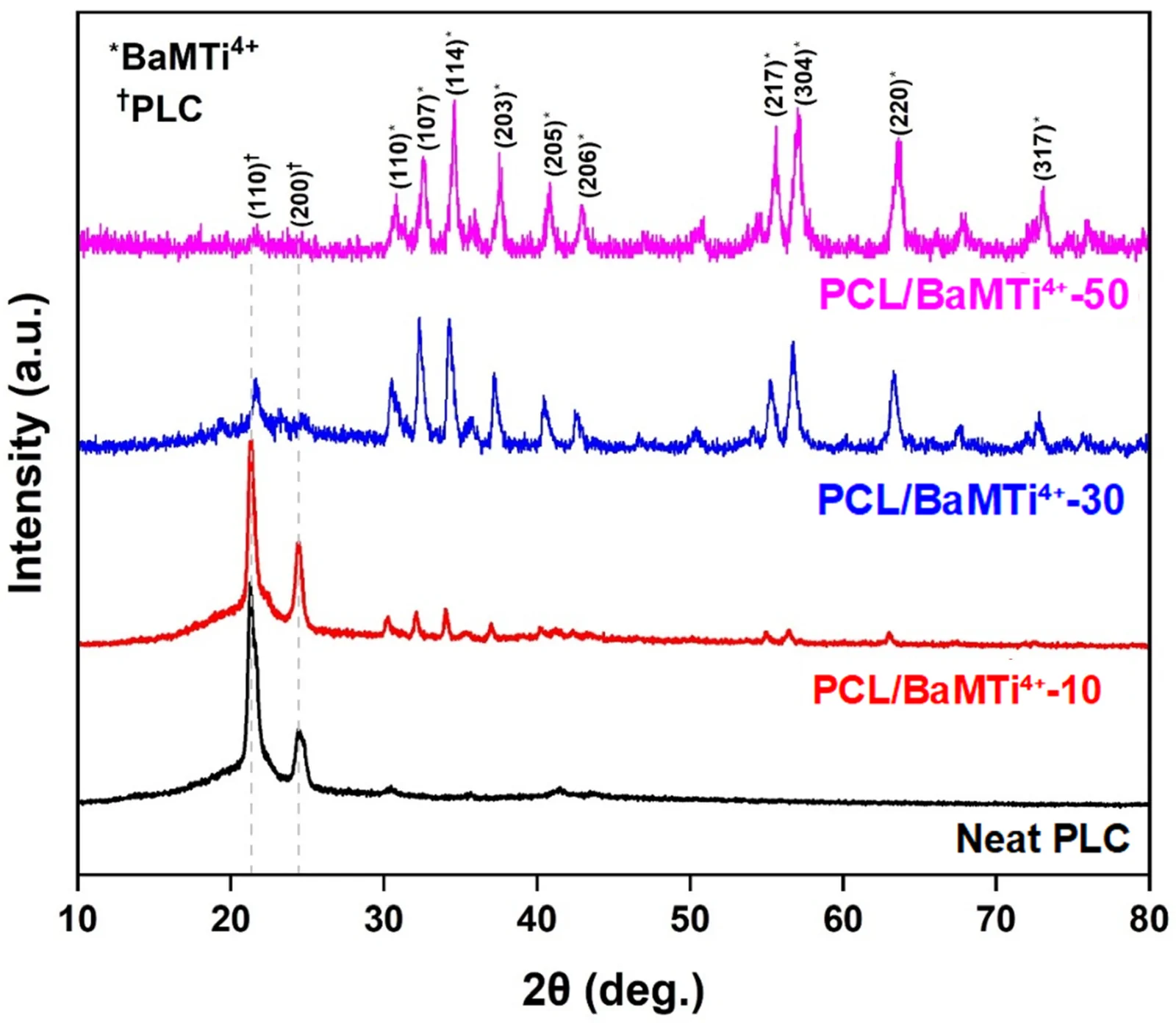
XRD patterns of neat PCL and PCL/BaMTi^4+^ composite films containing 10, 30, and 50 wt.% BaMTi^4+^. The dashed vertical lines indicate the characteristic PCL reflections at approximately 2θ = 21.5° and 23.8°, indexed to the (110) and (200) planes, respectively. Symbols identify reflections associated with BaMTi^4+^ (*) and PCL (†).

The X-ray diffraction patterns of neat PCL and PCL/BaMTi^4+^ composite films with increasing inorganic loading are shown in Figure 3. The neat PCL film displays two dominant reflections at 2θ~21.5° and 23.8°, assigned to the (110) and (200) planes of orthorhombic poly(ε-caprolactone) (PDF#24980-41-4). These crystalline reflections are superimposed on a broad diffuse contribution extending approximately over the 2θ = 15–30° region, consistent with the coexistence of ordered crystalline domains and a non-crystalline fraction characteristic of semicrystalline PCL. The preservation of the (110) and (200) reflections after solvent casting therefore confirms that crystalline PCL domains remain present in the films, while the underlying diffuse scattering reflects the non-crystalline component of the polymer matrix. Such coexistence is expected for semicrystalline PCL and is sensitive to processing conditions, including solvent evaporation, chain mobility, and polymer–filler interfacial constraints [14,16,37,44,45].

Upon incorporation of BaMTi^4+^, the diffraction profiles evolve from a polymer-dominated pattern toward a biphasic composite pattern in which the contribution of the crystalline inorganic phase becomes progressively more prominent. The ferrite reflections are consistent with the magnetoplumbite-type M-phase barium ferrite structure, as confirmed by ICSD card No. 259873 [46,47,48]. At 10% BaMTi^4+^, weak ferrite reflections appear superimposed on the intense PCL crystalline peaks, while the broad diffuse contribution associated with the non-crystalline polymer fraction remains clearly discernible. At 30 wt.%, well-defined BaMTi^4+^ reflections coexist with the PCL crystalline reflections, and the diffuse polymer contribution in the 15–30° region is still observable, confirming the coexistence of crystalline ferrite with the semicrystalline polymer matrix. At 50 wt.%, the diffraction pattern becomes strongly dominated by BaMTi^4+^ reflections, whereas both the characteristic PCL reflections and the underlying diffuse polymer contribution become comparatively less prominent. Importantly, the reduced visibility of the diffuse contribution at this composition should not be interpreted as evidence for the disappearance of the non-crystalline PCL fraction. Rather, the reduced polymer content, together with the much stronger X-ray scattering contribution of the Ba-containing crystalline phase, substantially decreases the relative contribution of PCL to the overall diffraction pattern. Therefore, the present XRD data establish the progressive increase in the relative diffraction contribution of BaMTi^4+^ with increasing filler content while preserving evidence of the semicrystalline nature of the PCL matrix at lower and intermediate loadings. Quantitative changes in the crystalline-to-non-crystalline fraction of PCL cannot be established from these diffraction patterns alone.

From a physical standpoint, the BaMTi^4+^ particles play a dual role in the crystallization of PCL. At low loading, the ferrite surface may act as a heterogeneous nucleation platform, reducing the free-energy barrier for local chain ordering and promoting the formation of crystalline nuclei at the polymer–ceramic interface. This behavior is commonly observed in semicrystalline polymer composites, where rigid inorganic surfaces provide energetically favorable sites for chain adsorption and crystallization [16]. However, as the ferrite concentration increases, the same interfaces that promote nucleation can also frustrate crystal growth. The polymer chains become increasingly confined between inorganic particles, and their segmental mobility is reduced during solvent evaporation. Under these conditions, nucleation may still occur, but lamellar growth becomes kinetically limited, leading to smaller, less perfect, or less abundant PCL crystalline domains. This competition between interface-induced nucleation and mobility-restricted crystal growth is central to understanding the non-linear structural evolution of polymer–ferrite composites [16,37]. The solvent-casting process further amplifies this competition. During solvent evaporation, the polymer concentration increases continuously, while particle–particle and particle–polymer distances decrease. This dynamic environment favors the development of concentration gradients, interfacial adsorption layers, and local confinement zones around BaMTi^4+^ particles. As a result, the final semicrystalline morphology is not determined only by the intrinsic crystallization tendency of PCL but also by the balance among solvent removal rate, polymer-chain relaxation, filler dispersion, and interfacial interaction strength. In this regard, the progressive dominance of BaMTi^4+^ reflections and the relative weakening of PCL peaks indicate a composition-driven reorganization of the composite microstructure, where the inorganic phase increasingly controls the diffraction response and constrains the polymer crystallization pathway. This interpretation agrees with broader physical descriptions of crystallization in confined and heterogeneous semicrystalline polymer systems [49,50]. The key structural outcome is not merely the presence of both phases, but the progressive shift in structural dominance from polymer-controlled crystallinity at low filler loading to ferrite-controlled diffraction at high loading. This transition reflects the combined effects of scattering contrast, inorganic dilution, heterogeneous nucleation, and interfacial restriction of polymer-chain mobility.

**Figure 4:**
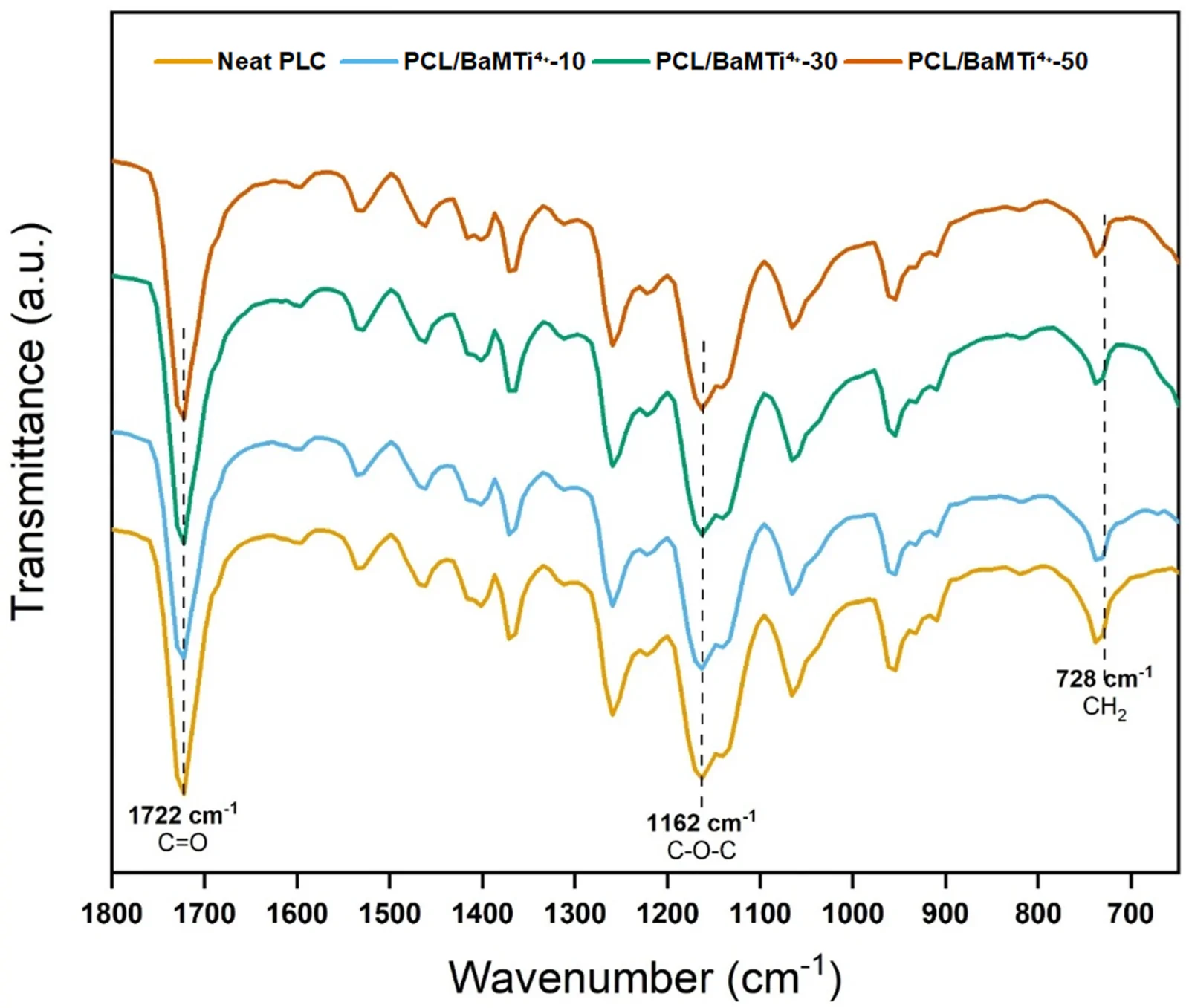
FTIR spectra of neat PCL and PCL/BaMTi^4+^ composite films prepared with different ferrite contents (10–50 wt.%), highlighting the vibrational signatures of the polymer matrix and ferrite phase.

Complementary information on the molecular structure of the PCL/BaMTi^4+^ composites is shown in Figure 4. In all compositions, the FTIR spectra are dominated by the characteristic vibrational fingerprint of PCL, demonstrating that the polymer backbone is chemically preserved after ferrite incorporation and solvent casting. The intense absorption band centered at approximately 1722 cm^−1^ is assigned to the stretching vibration of the ester carbonyl group, ν(C=O), while the band near 1162 cm^−1^ is associated with asymmetric stretching of the ester linkage, ν(C–O–C) [51,52,53].

The absorption at approximately 728 cm^−1^ is attributed to CH_2_ rocking vibrations, which are commonly related to the ordered methylene sequences in semicrystalline PCL domains [36,54,55]. The absence of new intense absorption bands indicates that BaMTi^4+^ incorporation does not cause detectable chemical degradation, chain scission, or covalent modification of the PCL matrix. This is relevant because the functional performance of these films depends on preserving the ester backbone while introducing an inorganic phase capable of modifying magnetic, dielectric, and interfacial properties. However, the progressive incorporation of BaMTi^4+^ induces subtle changes in the position, intensity, and profile of the PCL carbonyl and ester bands, suggesting modification of the local dipolar environment of the polymer chains. The C=O group is particularly sensitive to polymer–particle interactions due to its high polarity and strong dipole moment. Surface metal centers, oxygen vacancies, hydroxylated sites, or charged domains on BaMTi^4+^ particles may interact with PCL ester groups through dipole–dipole interactions, Lewis acid–base-type interactions, or electrostatic interfacial polarization. These local, non-covalent interactions can alter C=O and C–O–C vibrational energies by changing bond polarization and restricting segmental mobility near the particle surface. Ti^4+^ substitution may further modify ferrite surface charge distribution, defect chemistry, and acidity/basicity, influencing interactions with polar PCL groups. Thus, FTIR supports an altered interfacial environment, not new PCL–BaMTi^4+^ chemical phase formation. Variations in the ~728 cm^−1^ CH_2_ rocking band may also indicate perturbation of semicrystalline PCL chain packing. This interpretation is consistent with the XRD results, where the characteristic PCL reflections remain present but become progressively less dominant as the ferrite fraction increases. Thus, the FTIR and XRD data converge toward the same structural picture: the PCL phase is preserved, but its local ordering and chain mobility are increasingly affected by the presence of BaMTi^4+^ particles. These results show that PCL/BaMTi^4+^ films form biphasic polymer–ceramic composites, not reaction products. Their functionality arises from crystalline ferrite domains, semicrystalline PCL lamellae, and chemically preserved yet interfacially constrained polymer chains. Restricted chain mobility may affect stiffness and thermal relaxation, while polar PCL–ferrite interfaces promote interfacial polarization and dielectric or electromagnetic response. XRD-confirmed magnetoplumbite ferrite preservation remains essential for retaining the intrinsic magnetic contribution of the inorganic phase within composites. Similar structure–property relationships have been reported for magnetically active polymer composites, in which functional performance is controlled not only by filler content but also by filler dispersion, polymer crystallinity, and interfacial coupling [56,57].

### 3.2. Morphological Aspects

**Figure 5:**
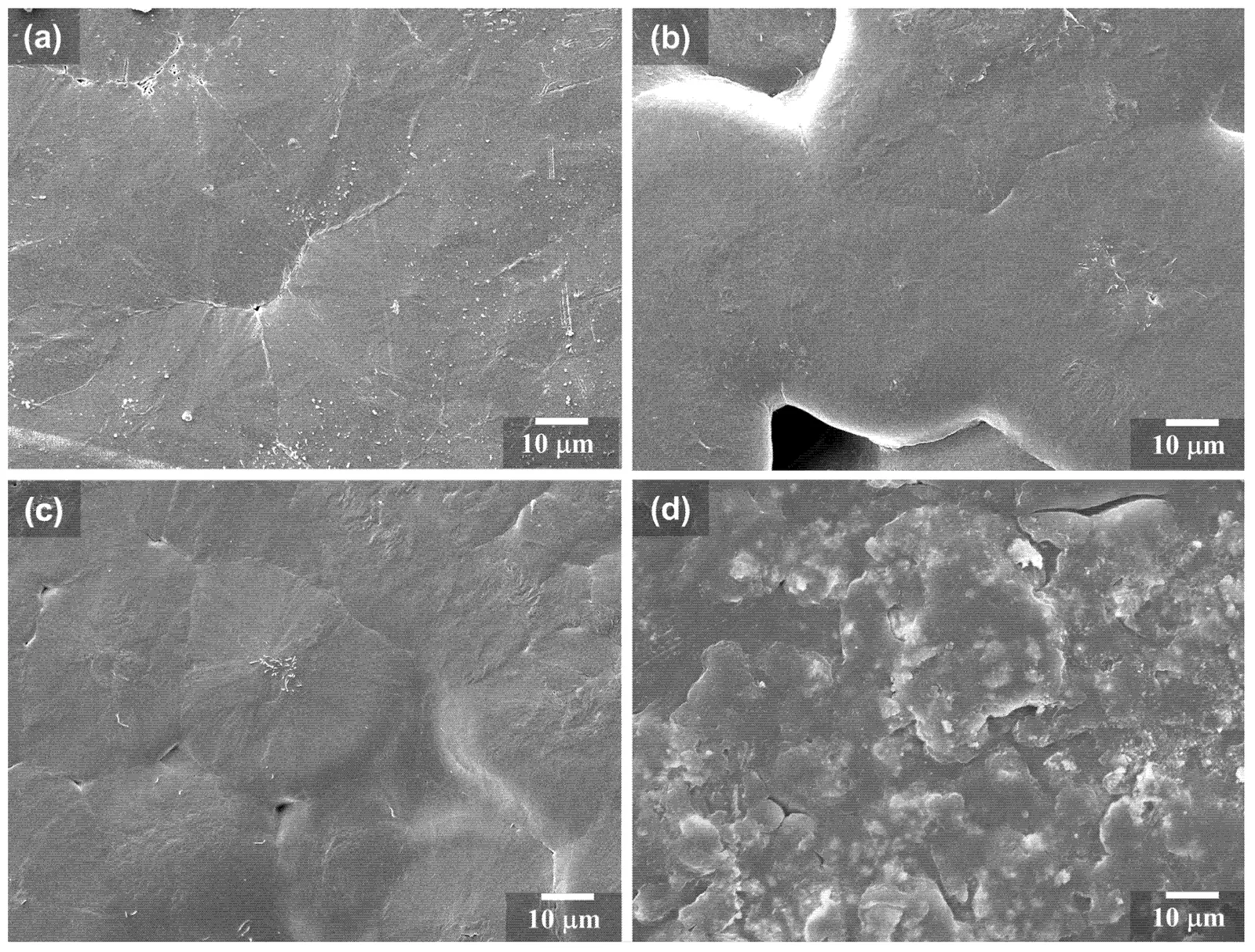
SEM micrographs of (**a**) neat PCL, (**b**) PCL/BaMTi^4+^-10, (**c**) PCL/BaMTi^4+^-30, and (**d**) PCL/BaMTi^4+^-50, showing the evolution of surface morphology with increasing BaMTi^4+^ content. Scale bars: 10 μm.

Following the structural and spectroscopic evidence for phase coexistence and chemical preservation of the polymer matrix, SEM analysis displayed in Figure 5 provides the mesoscale evidence needed to understand how the biphasic PCL/BaMTi4+ architecture is spatially organized.

The neat PCL film (Figure 5a), with an average thickness of 0.064 ± 0.015 mm, exhibits a relatively compact and smooth surface, with shallow radial marks and isolated microdefects that can be associated with solvent evaporation, shrinkage, and semicrystalline organization developed during film formation. After incorporation of 10 wt.% BaMTi^4+^ (Figure 5b), the average thickness increases to 0.117 ± 0.013 mm, while the surface remains predominantly polymer-rich but develops larger cavities and localized discontinuities. These features indicate that even relatively low ferrite loading perturbs the organization established during solvent casting, potentially through changes in solvent evaporation, polymer-chain packing, and local interfacial constraints. At 30 wt.% BaMTi^4+^ (Figure 5c), the film exhibits an average thickness of 0.158 ± 0.099 mm and a more heterogeneous morphology, characterized by visible depressions and increasingly irregular surface domains. At the highest ferrite loading (50 wt.%; Figure 5d), the average thickness is 0.119 ± 0.139 mm, and the surface undergoes a pronounced transition toward a rough, granular, and partially agglomerated morphology in which the ferrite-rich regions become increasingly evident. The substantially larger standard deviations observed for the 30 and particularly the 50 wt.% films indicate increasing spatial heterogeneity in film thickness at higher filler contents, consistent with the loss of morphological uniformity observed by SEM. Since film thickness and ferrite concentration were not independently controlled, the morphological evolution cannot be attributed exclusively to either parameter. Rather, the results suggest a coupled effect of increasing inorganic loading, local thickness variations, particle distribution, and polymer–filler interfacial organization during solvent casting. Similar behavior has been reported for PCL/metal and PCL/ceramic composites, in which inorganic fillers influence particle dispersion, polymer crystallization, and microstructural organization through local matrix–particle interactions [17]. At 50 wt.% BaMTi^4+^, the markedly irregular and partially agglomerated morphology is particularly relevant because increased filler proximity and connectivity may favor interfacial polarization and electromagnetic interactions, although excessive agglomeration may simultaneously introduce structural discontinuities and stress-concentration sites. This balance between filler dispersion, interfacial organization, and aggregation is consistent with previous ferrite/polymer systems, in which microstructural control plays an important role in determining the resulting functional response [4,58].

### 3.3. Topographical Spatial Investigation

**Figure 6:**
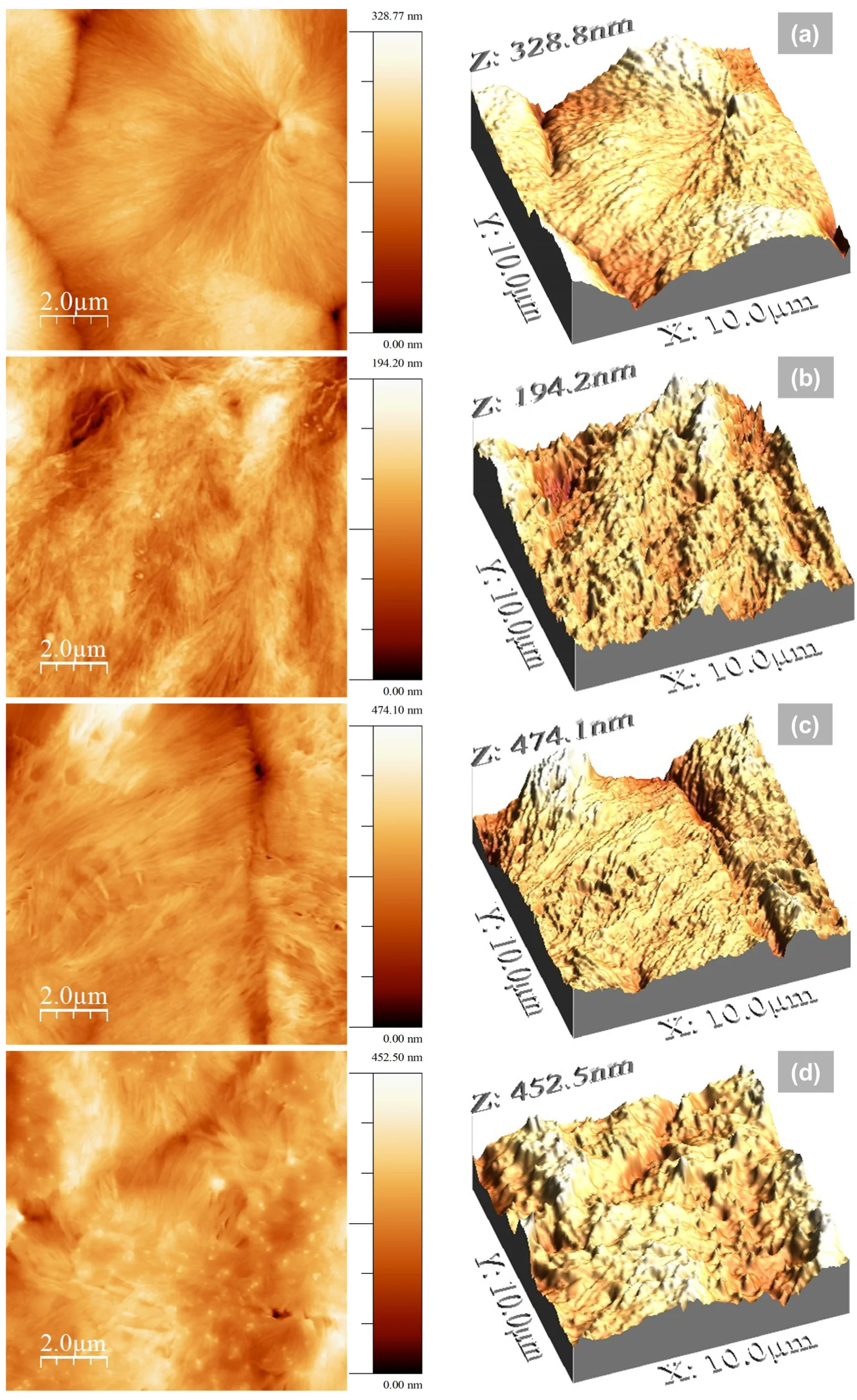
AFM images of composite films with increasing ferrite content: (**a**) neat PCL, (**b**) PCL/BaMTi^4+^-10, (**c**) PCL/BaMTi^4+^-30, and (**d**) PCL/BaMTi^4+^-50. Left: 2D topographic maps. Right: corresponding 3D surface reconstructions (scan area: 10 × 10 µm^2^), highlighting the progressive increase in surface roughness and morphological complexity with ferrite incorporation.

**Figure 7:**
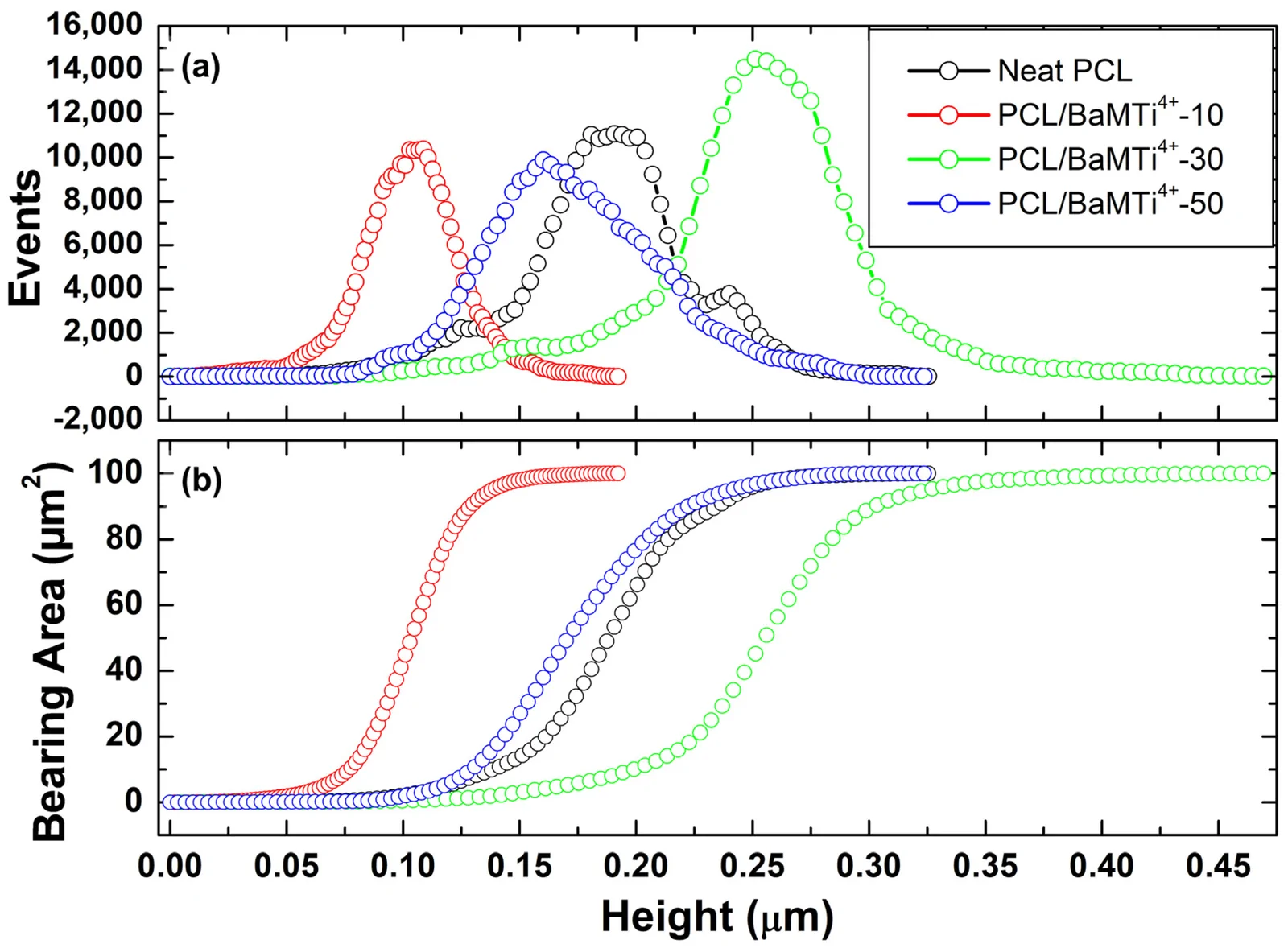
Height distribution (**a**) and Abbott–Firestone curves (**b**) of the film surfaces.

**Figure 8:**
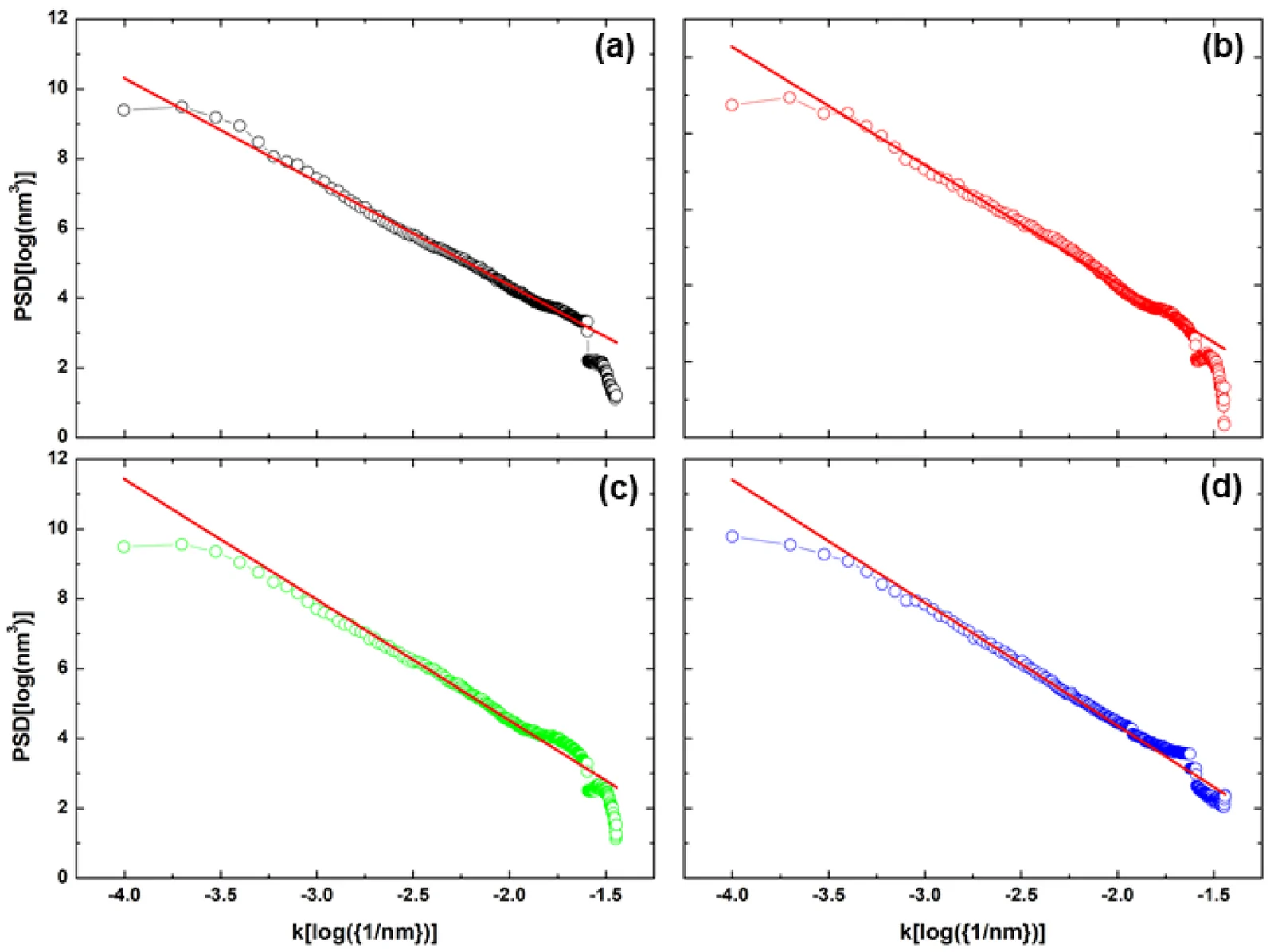
PSD analysis of AFM topographic data for PCL/BaMTi^4+^ films: (**a**) neat PCL, (**b**) PCL/BaMTi^4+^-10, (**c**) PCL/BaMTi^4+^-30, and (**d**) PCL/BaMTi^4+^-50. The variation in slope indicates changes in surface roughness and spatial organization across different length scales with increasing ferrite content.

Following the microstructural evidence of filler-induced surface reorganization, AFM analysis provides a quantitative nanoscale view of how BaMTi^4+^ modifies the surface architecture of the PCL matrix. Figure 6, Figure 7 and Figure 8, together with the ISO 25178-2 and monofractal parameters listed in Table 2, show that ferrite incorporation does not simply increase surface roughness; instead, it changes the physical regime of surface formation. The neat PCL film (Figure 6a) exhibits a relatively continuous semicrystalline texture with shallow radial domains, consistent with polymer-chain ordering during solvent evaporation. Its average roughness values, Sq = 33.68 nm and Sa = 25.94 nm (Table 2), indicate moderate nanoscale relief. Similar processing-dependent topographic features have been reported for PCL-based systems, where surface roughness is strongly affected by polymer organization and fabrication conditions, including chain mobility and solidification dynamics [13]. The PCL/BaMTi^4+^-10 film (Figure 6b) shows the lowest roughness, with Sq decreasing to 18.58 nm and Sa to 14.62 nm (Table 2). This is physically important because it suggests that low ferrite loading does not yet create a rough ferrite-dominated surface. Instead, the particles likely act as distributed nucleation or confinement centers, suppressing large-amplitude PCL surface relief and producing a more compact topography. This interpretation is supported by Figure 7b, which shows a smoother surface compared with neat PCL. Therefore, the 10% sample represents an interface-modified regime, where BaMTi^4+^ perturbs polymer crystallization without generating extensive particle-rich asperities. In the PCL/BaMTi^4+^-30 film (Figure 6c), the topography changes substantially. Sq increases to 41.22 nm and Sa to 30.93 nm (Table 2), while the AFM maps reveal stronger ridges, depressions, and heterogeneous surface domains. This indicates that the filler content has reached a threshold where polymer crystallization, particle–polymer interactions, and particle–particle proximity act together to produce a more developed surface. The PCL/BaMTi^4+^-50 film maintains this high-amplitude morphology, with Sq = 42.54 nm and Sa = 33.10 nm (Table 2), but Figure 6d suggests a more granular and ferrite-rich texture. Thus, the transition from 30% to 50% is not merely a linear roughness increase; it reflects a shift from polymer–filler co-organization to stronger inorganic-phase dominance. Comparable filler-dependent changes in polymer films have been reported in particle-reinforced composites, where filler dispersion strongly affects surface topography and micromechanical response [59].

The height distribution and Abbott–Firestone [60] curves in Figure 7 clarify the peak–valley architecture behind these roughness values. The neat PCL surface shows a slightly negative skewness, Ssk = −0.22 (Table 2), indicating a surface mildly dominated by valleys rather than sharp protruding peaks. The PCL/BaMTi^4+^-10 sample approaches symmetry, with Ssk = −0.11 (Table 2), consistent with its smoother and more compact surface. The PCL/BaMTi^4+^-30 film remains valley-skewed, Ssk = −0.26 (Table 2), suggesting deeper depressions associated with heterogeneous filler–polymer organization. In contrast, the PCL/BaMTi^4+^-50 film shifts to positive skewness, Ssk = 0.43 (Table 2), indicating the emergence of peak-dominated features, most likely associated with ferrite-rich asperities or particle aggregates. This change is directly reflected in the bearing area curves: the PCL/BaMTi^4+^-10 sample reaches full bearing area over a narrower height interval, while the PCL/BaMTi^4+^-30 and PCL/BaMTi^4+^-50 films require broader height intervals (Figure 7b), confirming a more vertically dispersed surface. The kurtosis values also provide useful information, but they must be interpreted carefully. All samples show Sku > 3, indicating height distributions with more pronounced extreme leptokurtic features than an ideal Gaussian surface [61,62,63]. However, because the table indicates no statistically significant difference for Sku by one-way ANOVA, this parameter should not be overused to claim a strong composition-dependent trend. The strongest and most reliable statistical evidence comes instead from the combined evolution of height-based parameters, the height distributions, and the bearing area curves. Together, these parameters show that BaMTi^4+^ loading controls not only the amplitude of roughness but also whether the surface is valley-dominated, symmetric, or peak-dominated.

The PSD analysis in Figure 8 adds a deeper length-scale interpretation and shows monofractal behavior in a delimited frequency domain. While Sa and Sq quantify vertical roughness amplitude, PSD and monofractal parameters describe how that roughness is spatially organized. The PSD slope α increases from 3.01 for neat PCL to 3.13, 3.49, and 3.62 for the 10%, 30%, and 50% composites, respectively (Table 2). This progressive steepening indicates that high-frequency roughness components decay more strongly as BaMTi^4+^ content increases. In physical terms, the surface becomes less controlled by fine, random nanoscale fluctuations and more dominated by larger, spatially correlated topographic features. This interpretation is reinforced by the Hurst exponent and fractal dimension. H increases from 0.51 for neat PCL to 0.81 for the PCL/BaMTi^4+^-50 film, while FD decreases from 2.49 to 2.19 (Table 2). Therefore, the composites become rougher in amplitude but less fractally jagged. This is a key result: higher ferrite loading increases the vertical scale of the surface, but the surface fluctuations become more persistent and spatially correlated. In other words, the PCL/BaMTi^4+^-30 and PCL/BaMTi^4+^-50 films are not simply “more irregular”; they display larger and more organized roughness domains. Recent fractal and PSD-based studies emphasize that rough surfaces cannot be adequately described by a single roughness number, because amplitude parameters and spatial frequency descriptors capture different physical aspects of surface formation [32,33].

Compared with recent reports on polymer, ceramic–polymer, and magnetic composite surfaces, our PCL/BaMTi^4+^ films provide a more physically resolved picture of how filler loading controls surface formation. Many studies still describe composite morphology mainly through average roughness or qualitative microscopy. However, the height-based and fractal descriptors allow the surface to be interpreted as a multiscale physical system. This is a stronger contribution because it distinguishes three regimes: a polymer-controlled semicrystalline surface in neat PCL, an interface-modified but relatively compact surface at PCL/BaMTi^4+^-10, and a highly developed multiscale topography at 30–50% loading. Recent AFM work on PCL-containing systems has shown that PCL surfaces can exhibit nanoscale roughness values and defects that depend strongly on processing conditions, polymer mobility, and solidification history. For example, melt-electrowritten PCL fibers showed measurable nanoscale roughness and surface imperfections linked to processing and crystallization effects [13]. In this regard, the present films are not merely rough PCL surfaces; they are magnetically active polymer–ceramic interfaces whose roughness is deliberately tuned by ferrite incorporation. This gives the work broader relevance than conventional PCL morphology studies. The results are also competitive with recent particle-reinforced polymer films, where increasing filler content commonly produces rougher surfaces with high peaks and deep valleys, while mechanical and functional behavior depends on the dispersion state of the filler [59]. Our approach shows that BaMTi^4+^ introduces not only topographic roughening but also a physically meaningful ferrite–polymer interface that may contribute to interfacial polarization, magnetic response, and electromagnetic attenuation. Recent reviews on polymer-based electromagnetic shielding composites stress that performance depends on the relationship among structure, filler type, matrix properties, and loss mechanisms, not on filler fraction alone [3]. Importantly, PCL/BaMTi^4+^-30 appears especially significant because it combines high topographic amplitude with strong multiscale organization, while the PCL/BaMTi^4+^-50 sample suggests the onset of ferrite-dominated aggregation. This distinction strengthens our results because it shows that the best surface architecture is not necessarily obtained at the highest filler content. Furthermore, recent discussions of polymer matrix composites for electromagnetic applications warn that excessive filler loading can promote agglomeration and deteriorate mechanical integrity, even when it improves functional connectivity [64].

## 4. Conclusions

This study demonstrates that solvent-cast PCL/BaMTi^4+^ films behave as composition-driven polymer–ferrite composites in which structural preservation, interfacial coupling, and multiscale surface organization evolve together. X-ray diffraction confirmed the coexistence of semicrystalline PCL and crystalline magnetoplumbite-type BaMTi^4+^, with the diffraction response progressively shifting from polymer-dominated at low filler content to ferrite-dominated at 50 wt.% loading. Importantly, the attenuation of PCL reflections at high BaMTi^4+^ content reflects not only scattering contrast and inorganic dilution but also the possible restriction of polymer-chain mobility and lamellar growth under interfacial confinement. FTIR-ATR analysis showed that the PCL backbone remains chemically preserved after ferrite incorporation, without evidence of chain scission, degradation, or formation of a new chemical phase. Subtle changes in the carbonyl and ester vibrational regions suggest local non-covalent interactions between polar PCL groups and surface-active sites of BaMTi^4+^, supporting a mechanism based on interfacial polarization and chain confinement rather than chemical transformation. SEM and AFM analyses revealed a clear morphology–topography transition with increasing ferrite content. The 10 wt.% composite produced the most compact surface, with reduced Sa and Sq values, whereas the 30 and 50 wt.% films developed higher-amplitude roughness and more heterogeneous ferrite-rich architectures. Height-distribution and Abbott–Firestone analyses further showed a transition from mildly valley-dominated surfaces to peak-dominated features at high loading. PSD and monofractal parameters confirmed that BaMTi^4+^ does not merely increase roughness; it reorganizes the surface into more spatially correlated domains, with increasing H and decreasing FD. Thus, this work establishes PCL/BaMTi^4+^ films as tunable magnetically active polymer–ceramic surfaces, where filler loading controls crystallinity, interfacial structure, morphology, and fractal topographical scaling.

## Figures and Tables

**Table 1:** Composition of neat PCL and PCL/BaMTi^4+^ composite films prepared by solvent casting. The BaMTi^4+^ loading is expressed as wt.% relative to the total solid content.

Sample	Chloroform (mL)	PCL (g)	BaMTi^4+^ (g)	Ferrite Content (wt%)
Neat PCL	5	0.5	0.0	0
PCL/BaMTi^4+^-10	5	0.45	0.05	10
PCL/BaMTi^4+^-30	5	0.35	0.15	30
PCL/BaMTi^4+^-50	5	0.25	0.25	50

**Table 2:** Surface height and monofractal parameters of neat PCL and PCL/BaMTi^4+^ composite films with increasing ferrite content, determined from AFM topographic data according to ISO 25178-2:2021. Sq: root-mean-square height; Sa: arithmetic mean height; Ssk: surface skewness; Sku: surface kurtosis; α: power spectral density slope; H: Hurst exponent; FD: fractal dimension.

Height Parameter	Neat PLC	PCL/BaMTi^4+^-10	PCL/BaMTi^4+^-30	PCL/BaMTi^4+^-50
Sq (nm)	33.68 ± 4.17	18.58 ± 3.36	41.22 ± 4.41	42.54 ± 4.26
Sa (nm)	25.94 ± 2.86	14.62 ± 2.36	30.93 ± 2.10	33.10 ± 3.92
Ssk	−0.22 ± 0.10	−0.11 ± 0.06	−0.26 ± 0.22	0.43 ± 0.30
Sku *	3.76 ± 0.29	3.34 ± 0.61	4.55 ± 1.51	4.01 ± 1.51
Fractal Parameter	Neat PLC	PCL/BaMTi^4+^-10	PCL/BaMTi^4+^-30	PCL/BaMTi^4+^-50
α	3.01 ± 0.06	3.13 ± 0.02	3.49 ± 0.04	3.62 ± 0.07
H	0.51 ± 0.03	0.57 ± 0.01	0.75 ± 0.02	0.81 ± 0.03
FD	2.49 ± 0.03	2.43 ± 0.01	2.25 ± 0.02	2.19 ± 0.03

* Samples without significant differences, one-way ANOVA (*p* < 0.05).

## Data Availability

The original contributions presented in this study are included in the article. Further inquiries can be directed to the corresponding author.
